# pycircdb: integrated circRNA database annotation for computational workflows

**DOI:** 10.1093/bioadv/vbag311

**Published:** 2026-10-15

**Authors:** Barry Digby, Stephen P Finn, Pilib Ó Broin

**Affiliations:** School of Mathematical and Statistical Sciences and Institute for Health Discovery and Innovation, University of Galway, Galway, H91 TK33, Ireland; Discipline of Histopathology, School of Medicine, Trinity College Dublin and Cancer Molecular Diagnostic Laboratory, Dublin, D08 RX0X, Ireland; School of Mathematical and Statistical Sciences and Institute for Health Discovery and Innovation, University of Galway, Galway, H91 TK33, Ireland

## Abstract

**Motivation:**

Currently, multiple databases exist for the annotation of circular RNAs, providing descriptions of their genomic features, FASTA sequences, predicted microRNA targets and RNA-binding protein interactions. Despite the availability of these resources, annotation remains cumbersome. Conflicting identifiers and genomic coordinates for circular RNAs across databases pose a challenge to reproducibility for users. Furthermore, current databases primarily provide access via web-based graphical user interfaces, bulk downloads and limited programmatic access, rendering their use in computational workflows challenging.

**Results:**

We present pycircdb, a Python command-line interface tool that accepts genomic coordinates of human circular RNAs as input, facilitating rapid queries of multiple curated circRNA databases for reproducible annotation. Each pycircdb database has been normalized to a common 0-based representation and cross-mapped between the hg19 and hg38 assemblies, accepting as input circular RNA coordinates from 0-based and 1-based sources using a tolerant matching strategy. pycircdb utilizes Apache Hamilton in conjunction with Parquet files, providing a modular, parallelized workflow with minimal disk space requirements.

**Availability and implementation:**

pycircdb is available for download via PyPI https://pypi.org/project/pycircdb under the GPL-3.0 license with source code and documentation available at https://github.com/BarryDigby/pycircdb and https://pycircdb.readthedocs.io/en/latest, respectively.

## 1 Introduction

Initially discovered in plant viroids (Sanger *et al*. 1976), yeast mitochondrial RNAs (Arnberg *et al*. 1980), and the hepatitis δ virus (Kos *et al*. 1986), circular RNAs (circRNAs) were believed to be by-products of aberrant splicing or intermediates discarded from intron-lariat debranching (Cocquerelle *et al*. 1993). Advancements in bioinformatics techniques and RNA-Sequencing protocols enabled the detection of circRNAs, with interest in the field invigorated when a landmark study by Salzman *et al*. identified circRNAs in hyperdiploid acute lymphoblastic leukemia diagnostic bone marrow samples (Salzman *et al*. 2012).

Originating from the same precursor messenger RNA as their linear RNA transcripts, circRNAs are formed through a process known as back-splicing in which a downstream 5′ splice donor site is reversely joined to an upstream 3′ splice acceptor site, forming a covalently closed loop structure (Jeck *et al*. 2013). Although the functions of most circRNAs have yet to be revealed, evidence suggests circRNAs harbor functionally active and evolutionarily conserved microRNA (miRNA) response elements within their mature spliced sequence (Hansen *et al*. 2013), suggesting a regulatory role within the competing endogenous RNA network by sequestering endogenous miRNAs (Denzler *et al*. 2014). Furthermore, circRNAs can form a scaffold with RNA-binding protein(s) (RBP), acting as modulators of transcription and splicing, and as templates for translation (Shi and Zhao 2025).

Due to their circular topology, circRNAs lack a poly(A) tail and 5′ and 3′ ends, conferring resistance to RNase degradation, resulting in a stable RNA structure with a higher half-life compared to their linear counterparts (Salzman *et al*. 2012). Several studies have identified circRNAs in human biofluids, including saliva, plasma, serum, and in exosomes, making them attractive candidate biomarkers for non-invasive liquid biopsy approaches (Memczak *et al*. 2015). circRNAs further exhibit stage and tissue-specific expression (Gruner *et al*. 2016) and are dysregulated across a wide range of cancers (Conn *et al*. 2024).

These discoveries have motivated the development of numerous bioinformatics pipelines for circRNA detection, and in turn, dedicated databases have emerged to consolidate the circRNAs reported by these pipelines across human studies (Digby *et al*. 2024). Whilst these databases are valuable resources, they suffer from a lack of a standardized naming convention for their circRNA identifiers. For example, the hg19 circRNA chr1:27087346–27089776|+ returns the following identifiers: ASCRP3003018 (Arraystar), hsa-ARID1A_0002 (circAtlas), hsa_ARID1A_0003300 (circBank), hsa_circ_0006913 (circBase). Compounding this problem is the fact that individual databases are built against different reference genome assemblies and adopt inconsistent coordinate conventions, mixing 0-based and 1-based systems. Most significantly, a subset of databases support only single circRNA queries, making systematic annotation of experimentally detected circRNAs an arduous process.

No existing resource addresses all three barriers (Table 1, available as supplementary data at *Bioinformatics Advances* online). Individual databases provide web-based interfaces or bulk downloads, but none offers a unified programmatic interface that queries multiple databases concurrently, normalizes coordinates across genome assemblies and base conventions, and returns results in a format directly consumable by downstream analysis scripts. BioMart (Durinck *et al*. 2009) provides general genomic annotation but has not yet integrated circRNA-specific resources; circAtlas (Dudekula *et al*. 2016) in addition to other databases, supports only single-query submissions; the CircFunBase API accepts only single circRNA queries; and bulk-download files from individual databases still require users to independently reconcile conflicting identifiers and coordinate systems before any cross-database comparison is possible.

**Table 1:** Summary of circRNA databases integrated in pycircdb and their use in corresponding modules.^a^

Database	Reference	Coordinates	Version	Accessed	Entries	Annotation	FASTA	miRNA	RBP
**Arraystar**	hg19	1-based	2.0	January 2025	13 154	**✓**	**✓**	**✗**	**✗**
**circAtlas**	hg38	1-based	3.0	January 2026	768 986	**✓**	**✗**	**✗**	**✗**
**circBank**	hg19	1-based	1.0	January 2026	828 802	**✓**	**✓**	**✗**	**✗**
**circBase**	hg19	0-based	1.0	January 2026	92 369	**✓**	**✓**	**✗**	**✗**
**CircNet**	hg19	1-based	2.0	January 2026	6 913 950	**✗**	**✗**	**✓**	**✗**
**CIRCpedia**	hg38	0-based	3.0	October 2025	849 495	**✓**	**✓**	**✗**	**✗**
**circRNADb**	hg19	1-based	1.0	March 2026	32 914	**✓**	**✓**	**✗**	**✗**
**CSCD**	hg38	1-based	2.0	January 2026	2 749 897	**✓**	**✓**	**✓**	**✓**
**exoRBase**	hg38	1-based	3.0	May 2026	126 816	**✓**	**✗**	**✗**	**✗**

a “Entries” is the total number of circRNA records in each pycircdb annotation lookup table. CSCD provides 177 600 389 circRNA-miRNA and 106 438 146 circRNA-RBP entries, respectively. For databases with circRNA sequences (FASTA module), the number of records is identical to “Entries” and is therefore not repeated. Liftover failure and one-to-many mapping counts per database are reported in Table 2, available as supplementary data at *Bioinformatics Advances* online.

We present pycircdb, a Python tool that takes as input hg19 or hg38 circRNA coordinates from multiple samples and performs a lookup of nine manually reformatted circRNA databases, returning circRNA annotations, FASTA sequences, and precomputed miRNA and RBP interactions (Fig. 1). As a command-line interface (CLI) tool, pycircdb can be integrated into computational workflows as part of a quantification pipeline or used as a post-hoc tool to query circRNAs. pycircdb is open-source, with source code freely available at https://github.com/BarryDigby/pycircdb.

**Figure 1:**
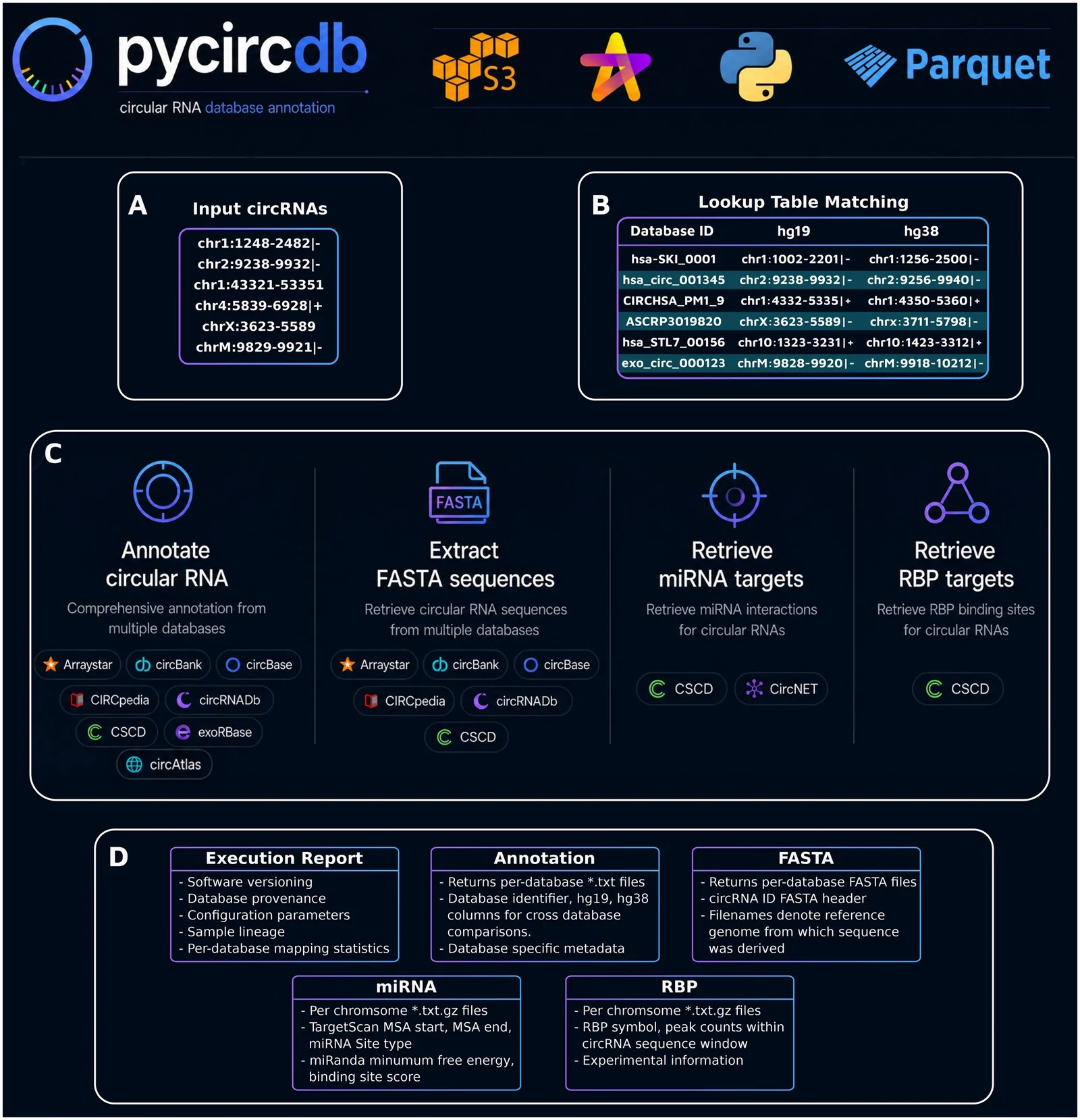
Overview of the pycircdb Python tool. (A) Input hg19 circRNAs in chr: start-end|strand format provided in a single-column text file (strand optional, configuration file not shown). (B) Perform matching against pycircdb lookup tables. Exact matching (chr2:9238–9932|-), strand ambiguous matching (chrX: 3623–5589) and tolerant coordinate matching (chrM: 9829–9921|-). (C) Lookup hits are used as inputs to the annotate,fasta,miRNA and rbp modules. (D) Summary of output files generated by pycircdb.

## 2 Implementation

### 2.1 Database design

Publicly available human hg19/hg38 circRNA database files from Arraystar, circAtlas (Wu *et al*. 2020), circBank (Liu *et al*. 2019), circBase (Glažar *et al*. 2014), CircNet (Chen *et al*. 2022), CIRCpedia (Dong *et al*. 2018), circRNADb (Chen *et al*. 2016), CSCD (Feng *et al*. 2022), and exoRBase (Lai *et al*. 2022) were downloaded from their respective sources (Table 1). Reformatting of each database was performed manually, encompassing conversion of 1-based genomic coordinates to a common 0-based representation and cross-mapping between hg19 and hg38 using UCSC liftOver (Hinrichs *et al*. 2006) with the hg19ToHg38.over.chain.gz and hg38ToHg19.over.chain.gz chain files at default settings. CSCD records required additional handling. Briefly, CSCD records detected by CIRI and circRNA_finder can have multiple entries for a record: (i) a circRNA record with ≥2 quantification tools as evidence, of which CIRI and/or circRNA_finder are members; (ii) the same record reported on the antisense strand with only circRNA_finder as evidence; and (iii) a 1-based version of the record supported by only CIRI. We removed the 1-based CIRI entries and circRNA_finder antisense entries to prevent duplicate annotation records in the pycircdb output. The removal of antisense circRNA_finder entries is validated by the circBase identifiers retained in CSCD: since circBase identifiers are strand-specific, a circBase ID cannot legitimately annotate entries on opposing strands. We therefore applied the same filtering logic to analogous CIRI and circRNA_finder entries lacking a circBase identifier. During the conversion from hg19 to hg38, segmentally duplicated and repetitive-rich regions within the genome produced “one-to-many” hg19 to hg38 coordinates, which are accounted for in pycircdb by retaining multi-mapped coordinates rather than collapsing identifiers to one region (Fig. 1, available as supplementary data at *Bioinformatics Advances* online); the number of records successfully mapped, failing liftover, and producing one-to-many mappings is reported per database in Table 2, available as supplementary data at *Bioinformatics Advances* online.

**Table 2:** pycircdb benchmarking results.^a^

Input	Annotation	FASTA	miRNA	RBP	Wall clock (s)	Peak RAM (GB)	Results disk (MB)
**100^†^**	96	96	96	96	13.7	2.3	2.6
**1000^†^**	974	974	964	962	16.2	2.5	23.4
**10 000^†^**	9789^(7)^	9789	9685	9685	25.8	2.7	228.8
**100 000**	14 123^(30)^	14 104	13 273	13 223	35.0	3.1	240.5
**200 000**	28 307^(45)^	28 271	26 368	26 241	52.9	3.3	469.8
**300 000**	42 530^(63)^	42 476	39 298	39 108	66.7	3.8	690.7

a Inputs marked

† are high-confidence circRNA candidates (≥3 back-splice junction reads, detected by ≥3 of seven quantification tools); remaining inputs are circRNAs with no filtering applied for performance scaling assessment. Annotation and FASTA columns report unique circRNAs across results (rate of annotation multi-mapping circRNAs are in superscript); miRNA and RBP report unique hg38 coordinates with miRNA/RBP interactions. Database files (3.6 GB) were pre-cached; download time is excluded.

Next, lookup tables were constructed for each database containing the original database identifier alongside the corresponding hg19 and hg38 coordinates (Figs 2 and 3, available as supplementary data at *Bioinformatics Advances* online; Tables 3 and 4, available as supplementary data at *Bioinformatics Advances* online). The lookup tables are used to match user-provided circRNA inputs and serve as the basis for querying the annotation, FASTA, miRNA, and RBP database tables based on user-supplied commands supplied at runtime.

Annotation, miRNA and RBP tables were modified to include both hg19 and hg38 lookup keys in addition to their original columns, whilst FASTA tables were reformatted to a two-column format containing the original database identifier and the circRNA sequence. FASTA sequences were retained in their original reference genome sequence; Arraystar, circRNADb, circBank, and circBase are hg19-based, while CIRCpedia and CSCD are hg38-based. Sequences are retrieved exactly as provided by the original databases and are not regenerated from input coordinates. The reference assembly is encoded in each output filename (e.g. arraystar_hg19.fasta, cscd_hg38.fasta), making the source assembly unambiguous without requiring cross-reference to external documentation. Summaries of shared circRNA coordinates in FASTA database tables are available in Fig. 4, available as supplementary data at *Bioinformatics Advances* online and Table 5, available as supplementary data at *Bioinformatics Advances* online, respectively.

All lookup, annotation, sequence, miRNA and RBP tables are stored as Apache Parquet files in a publicly accessible AWS S3 bucket. The complete database occupies 3.6 GB of disk space; however, pycircdb only downloads the files required for a given run—those corresponding to the databases specified by the user and, for the miRNA and RBP resources, only the chromosome tables matching the chromosomes present in the input data.

Databases were selected on the basis of publicly available human data for bulk download. Several candidate resources were evaluated but excluded: CircInteractome was omitted because its circRNA set overlaps substantially with circBase without providing additional annotation; CircFunBase was excluded owing to its limited human entry count (3799 records at the time of access); and multiple databases were inaccessible (Table 1, available as supplementary data at *Bioinformatics Advances* online). Databases providing only sample-level membership information without coordinate-level annotation (MiOncoCirc, NeuroCirc) were also excluded as they would not enrich pycircdb outputs. Database files are versioned independently of the pycircdb package using a db_version.txt file shipped with the package. This file specifies the current database version and is used to resolve the correct S3 directory at runtime (e.g. v1_0/for database version 1.0, v1_1/for 1.1). Prior database snapshots are retained on S3 indefinitely, ensuring that any past release of pycircdb continues to query the same database it was originally distributed with. Database updates are communicated via GitHub releases and a version badge in the repository README.

To confirm that coordinate-based lookup correctly resolves database-specific identifiers, the six circRNAs comprising pycircdb’s init-demo demonstration dataset—each present in all eight annotation databases—were queried; pycircdb returned the expected identifier in every database without exception (Table 6, available as supplementary data at *Bioinformatics Advances* online).

### 2.2 pycircdb design

At its core, pycircdb utilizes Apache Hamilton, a declarative framework that maps dependencies between individual Python functions to construct a directed acyclic graph (DAG). This approach facilitates configuration-driven, parallel execution of the DAG code. pycircdb’s codebase is composed of several modules: (i) ingest user inputs, download all lookup tables and return lookup hits; (ii) download the requisite annotation, FASTA, miRNA and RBP files based on user command parameters, and for database files split by chromosome, downloading only the partitions corresponding to chromosomes represented in the lookup hits; (iii) pool hg19/hg38 circRNA coordinates from each lookup table for use in downstream subsetting steps; (iv) subset the annotation database tables and write to files; (v) subset the FASTA database tables using pooled circRNA coordinates and their corresponding database identifiers and write to files; (vi) subset the miRNA database tables and write to gzip-compressed files; and (vii) subset the RBP database tables and write to gzip-compressed files.

Given that circRNA quantification tools report coordinates in either 0-based or 1-based outputs and, in some cases, omit strand information, pycircdb applies a tolerant coordinate matching strategy to accommodate the output of any quantification tool run on human samples once converted to the expected format. For each input circRNA, three candidate start positions are generated (original, −1 bp, +1 bp) whilst the end coordinate is held fixed, capturing the single-base offset introduced by 0-based versus 1-based conventions. When strand is provided, matching is performed exactly against the database coordinate including strand; when strand is omitted, the strand suffix is stripped from database entries prior to matching, and all strand information at matching positions is returned. Users need only consolidate the chromosome, start, end and (optionally) strand into pycircdb’s chr: start-end|strand format for inputs. Chromosome names require the chr prefix; supported chromosomes are chr1–chr22, chrX, chrY and chrM. chrM is supported by the annotate and fasta commands but excluded from the mirna and rbp modules, as chromosome-level predictions for chrM are absent from the source databases.

Each matched entry produces one output row per circRNA-database pair in the designated output “database hits” file; a single input circRNA may return multiple rows where it maps to multiple coordinates via one-to-many liftover. Currently, pycircdb documents multi-mapping circRNAs in the execution report summary file, however, coordinate tolerant matches and strand-ambiguous matches are not distinguished in outputs.

### 2.3 pycircdb invocation

pycircdb is configured via a JSON file, specifying both global and sample-level parameters for a workflow run.

Global parameters include: (i) max_tasks: the number of concurrent threads for Apache Hamilton parallelism (default: 1); (ii) output_dir: the output directory to write results to using relative or full paths (default: results/); and (iii) tmp_dir: the directory used to store downloaded database files. When running on Cloud/HPC, we recommend using a shared storage space accessible to other users for subsequent runs (default: tmp/).

Sample parameters can be specified multiple times under the samples parent key. Each child collection includes the sample name used to name the subdirectories of the output directory, the file_path parameter providing the relative or full path to the input sample file containing circRNAs and the reference parameter specifying the reference genome used to generate the input circRNAs (hg38 or hg19).

pycircdb contains global click options, click commands and click command-level options. Global options include -c/—config denoting the configuration file, which is mandatory. The -v/—verbose option is set to 0, 1 and 2, respectively, with higher integers representing more verbose output. Five commands are available to users: (i) init-demo: creates a minimal test configuration file complete with a toy dataset; recommended for first-time users to inspect outputs generated by pycircdb; (ii) annotate: return all database hits from Arraystar, circAtlas, circBank, circBase, CIRCpedia, circRNADb, CSCD and exoRBase, providing comprehensive information of circRNAs; (iii) fasta: return all predicted circRNA sequences from Arraystar, circBank, circBase, CIRCpedia, circRNADb and CSCD; (iv) mirna: retrieve precomputed circRNA-miRNA interactions from CircNet and CSCD, generated using miRanda, PITA and TargetScan; and (v) rbp: retrieve precomputed circRNA-RBP interactions from CSCD database. Both the annotate and fasta commands accept the command-level option -d/—database, where users can specify specific databases to query instead of the default of all databases. The mirna command accepts the option -a/—algorithm, permitting users to subset circRNA-miRNA results to miRanda, PITA or TargetScan, or any combination thereof (Table 7, available as supplementary data at *Bioinformatics Advances* online).

### 2.4 pycircdb outputs

pycircdb produces five categories of output, all written to a user-specified directory.

*Execution report.* A plain-text execution report (execution_report_<timestamp>.txt) is generated after every run, recording the run timestamp, software and database bundle versions, the configuration used, per-sample inputs and reference genomes, the databases and miRNA prediction algorithms selected, and database provenance (native genome build, version, and access date). Per-database mapping statistics are provided as the ratio of input to database hits (one-to-one). Multi-mappers, if present, are also reported.

*Annotation.* For each selected annotation database (Arraystar, circAtlas, circBank, circBase, CIRCpedia, circRNADb, CSCD, and exoRBase) a tab-delimited file is written containing the database-native circRNA identifier, coordinates in both hg19 and hg38, and database-specific metadata. Common fields across databases include chromosomal coordinates, strand, host gene symbol, circRNA biotype (exonic, intronic, intergenic, or exon-intron circRNA), and transcript of origin where available. CSCD additionally reports subcellular localization, detection algorithms, read counts per sample, alternative splicing events, and circular-to-linear expression ratios.

*FASTA sequences.* For databases that provide spliced circRNA sequences (Arraystar, circBank, circBase, CIRCpedia, circRNADb, and CSCD), a FASTA file is written containing the circular RNA sequence for each circRNA.

*miRNA interactions.* Predicted miRNA binding sites are reported in a per-chromosome *.txt.gz tab-delimited file aggregating results from CircNet and CSCD across the miRanda, PITA, and TargetScan algorithms, with columns for binding site coordinates, site type, score, and minimum free energy where available.

*RBP interactions.* RBP interactions derived from CSCD are reported in a per-chromosome *.txt.gz tab-delimited file containing the RBP symbol, binding site coordinates, number of RBPs predicted to bind at the site coordinates and tissue context (normal, cancer, or common).

A full description of pycircdb outputs, including detailed descriptions of each database column, is provided in the documentation: https://pycircdb.readthedocs.io/en/latest/outputs.html.

### 2.5 pycircdb testing and performance

pycircdb is tested via a cross-platform continuous integration (CI) pipeline on GitHub Actions, executing all five commands (init-demo, annotate, fasta, mirna and rbp) across Ubuntu, macOS, and Windows using Python 3.10–3.14. Benchmarking was performed using circRNA candidates from two human glioblastoma RNA-seq samples (SRR5133906 and SRR5133907) detected with seven quantification tools: CIRCexplorer2, CIRIquant, DCC, find_circ, circRNA_finder, MapSplice and Segemehl. Annotation yield was evaluated on high-confidence inputs of 100, 1000 and 10 000 circRNAs [≥3 back-splice junction reads, detected by ≥3 tools independently (Digby *et al*. 2023)], returning annotations for >97% of candidates across all four modules. Runtime and memory scaling were assessed on randomly sampled inputs of 100 000–300 000 circRNAs; pycircdb processed 300 000 circRNAs across all four modules in 66 s at a peak of 3.8 GB RAM, with database files pre-cached locally (Table 2).

## 3 Conclusion

We have developed a Python package enabling rapid, CLI access to multiple publicly available circRNA databases, addressing the ambiguous nomenclature and absence of programmatic interfaces that currently limit their utility. pycircdb is currently limited to human circRNAs based on hg19 and hg38 assemblies; extension to additional species and genome assemblies represents a natural avenue for future development. Given the modular architecture of pycircdb’s codebase, we anticipate that additional databases will be incorporated as the field expands. Furthermore, Chen *et al*. have proposed a standardized nomenclature for circRNAs (Chen *et al*. 2023); pycircdb’s capacity to reconcile annotations across existing databases positions it as a practical resource for translating current catalogues to this emerging standard.

## Supplementary Material

vbag311_Supplementary_Data

## Data Availability

All database files discussed in the manuscript are available for download in Parquet format by running pycircdb. Benchmarking scripts are available on GitHub under the benchmarking/performance directory with a seed for reproducibility.
