# Supplementary material for: pycircdb: integrated circRNA database annotation for computational workflows
Source: Bioinform Adv. 2026 Oct 15;6(1):vbag311. doi: 10.1093/bioadv/vbag311 (PMC13721112; doi:10.1093/bioadv/vbag311)
Supplement: Supplementary file 1 [file vbag311_supplementary_data.pdf]

Supplementary Materials

pycircdb: integrated circRNA database annotation for computational workflows

Barry Digby, Stephen P. Finn, Pilib Ó Broin

October 4, 2026

Table 1: Feature comparison of circRNA databases supporting circRNAs in *Homo sapiens*

Resource	Annotation	FASTA	miRNA	RBP	API	Batch queries	Multiple species
circAtlas [1]	✓	✓	✓	✓	✗	✗	✓
circBase [2]	✓	✓	✗	✗	✗	✓	✓
CircBank [3]	✓	✓	✓	✗	✗	✗	✓
CircInteractome [4]	✓	via circBase	✓	✓	✗	✗	✗
CircNet [5]	✓	✓	✓	✓	✗	✗	✗
CircFunBase [6]	✓	✗	✗	✗	✗	✗	✓
CIRCpedia [7]	✓	✓	✗	✗	✗	✓	✓
CSCD [8]	✓	✓	✓	✓	✗	✗	✗
circRNADb [9]	✓	✓	✗	✗	✗	✗	✗
CircRiC† [10]	—	—	—	—	—	—	—
Circ2Disease† [11]	—	—	—	—	—	—	—
CircR2Disease† [12]	—	—	—	—	—	—	—
Circ2Traits† [13]	—	—	—	—	—	—	—
circRNADisease [14]	✓	✗	✓	✗	✗	✗	✓
DeepBase† [15]	—	—	—	—	—	—	—
exoRBase [16]	✓	✗	✗	✗	✗	✗	✗
MiOncoCirc [17]	✗	✗	✗	✗	✗	✗	✗
NeuroCirc [18]	✓	✗	✗	✗	✗	✗	✗
starBase [19]	✓	via circBase	✓	✓	✓	✓	✓
TRCirc† [20]	—	—	—	—	—	—	—
**pycircdb**	✓	✓	✓	✓	✓	✓	✗

†Indicates the database was not available at the time of drafting the manuscript.

Direction	Input	Output
hg19→hg38	`chr10:47121120-47122996`|−	`chr10:46426759-46428634`|+
hg38→hg19	`chr10:46426759-46428634`|+	`chr10:47121120-47122996`|−
hg38→hg19	`chr10:47956026-47957901`|−	`chr10:47121120-47122996`|−

(A)

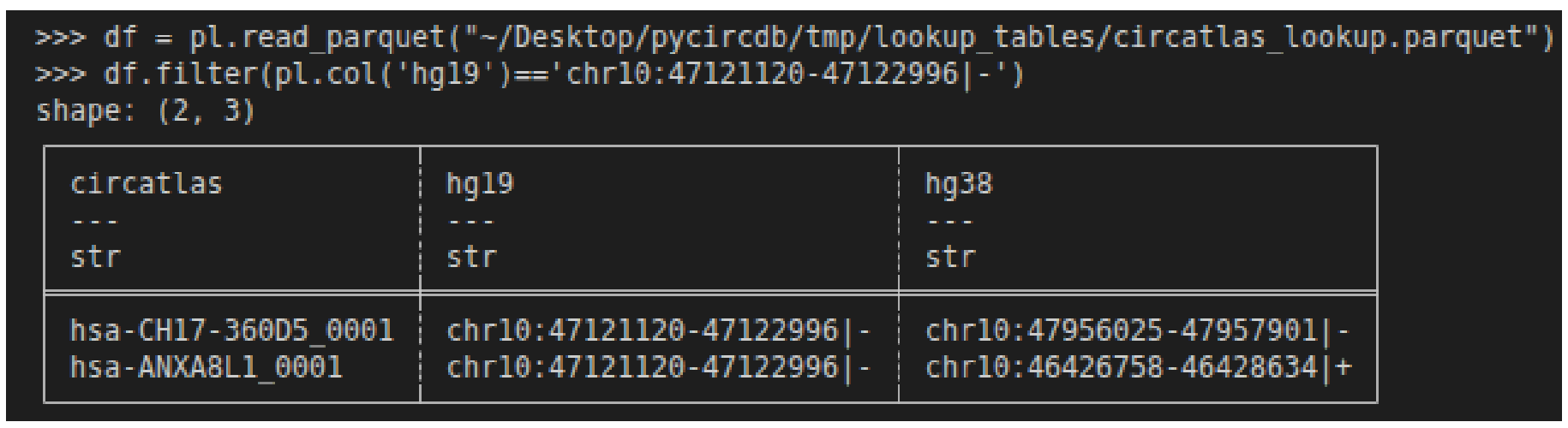

```
>>> df = pl.read_parquet("~/Desktop/pycircdb/tmp/lookup_tables/circatlas_lookup.parquet")
>>> df.filter(pl.col('hg19')=='chr10:47121120-47122996|-')
shape: (2, 3)
```

circatlas --- str	hg19 --- str	hg38 --- str
hsa-CH17-360D5_0001	chr10:47121120-47122996|-	chr10:47956025-47957901|-
hsa-ANXA8L1_0001	chr10:47121120-47122996|-	chr10:46426758-46428634|+

(B)

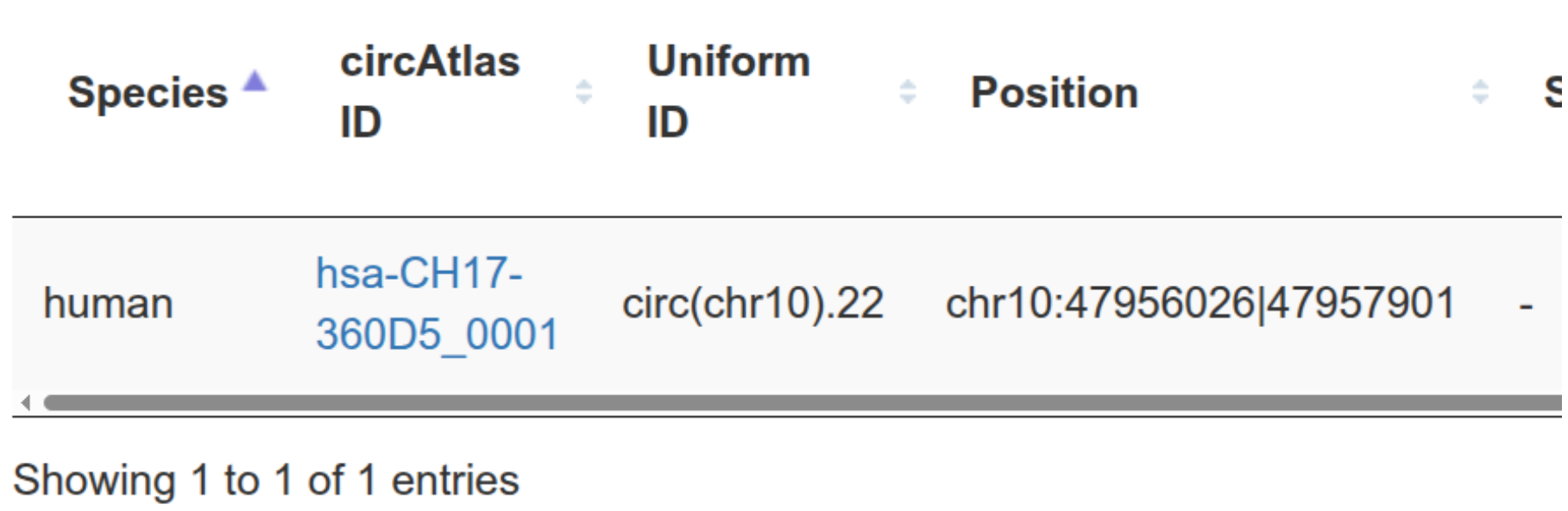

Species	circAtlas ID	Uniform ID	Position	S
human	hsa-CH17-360D5_0001	circ(chr10).22	chr10:47956026|47957901	-

Showing 1 to 1 of 1 entries

(C)

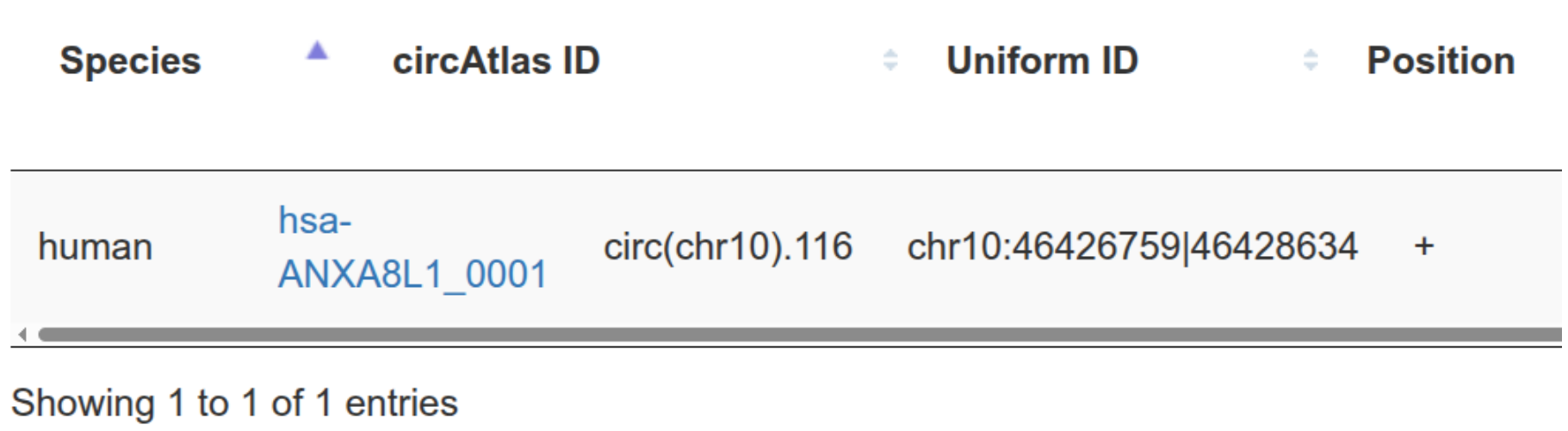

Species	circAtlas ID	Uniform ID	Position	
human	hsa-ANXA8L1_0001	circ(chr10).116	chr10:46426759|46428634	+

Showing 1 to 1 of 1 entries

(D)

Figure 1: One-to-many hg38 mapping for the hg19 locus chr10:47121120-47122996|−. (A) UCSC liftOver results for the locus. The forward liftover (hg19→hg38) returns a single hg38 coordinate, even with the `-multiple` flag. However, the reverse liftover (hg38→hg19) shows that two independent hg38 coordinates each map back to the same hg19 coordinate, reflecting a segmental duplication present in hg38 but collapsed in hg19. pycircdb captures this asymmetry by running the reverse lift-over from each hg38-native database entry; both mappings are retained rather than collapsed. Note: UCSC liftOver and circAtlas display coordinates in 1-based convention; pycircdb normalises all coordinates to 0-based. (B) pycircdb lookup table retaining both hg38 mappings under the same hg19 key. (C) circAtlas entry for hsa-CH17-360D5_0001, confirming the first hg38 mapping (chr10:47956026-47957901|−). (D) circAtlas entry for hsa-ANXA8L1_0001, confirming the second hg38 mapping (chr10:46426759-46428634|+).

Table 2: Lookup table breakdown between hg19 and hg38 coordinates.

Database	Total	Unique hg19	Unique hg38	hg19-only	hg38-only	Multi-mapped
Arraystar	13,154	13,154	13,142	12	0	0
circRNADb	32,914	32,914	32,865	38	0	11
circAtlas	768,986	767,654	768,986	0	1,311	21
circBank	828,802	828,802	828,289	478	0	35
circBase	92,369	92,369	92,079	258	0	32
CIRCpedia	849,495	847,230	849,495	0	1,928	337
CSCD	2,749,897	2,745,905	2,749,897	0	3,863	129
exoRBase	126,816	126,360	126,816	0	378	78

Reconciling hg19 and hg38 coordinate counts

Each pycircdb lookup table stores every circRNA in both hg19 and hg38 genome builds. The number of distinct coordinates is not identical between builds, for two independent reasons captured in Supplementary Table 2. First, some entries carry a coordinate in only one build (hg19-only or hg38-only) - these are circRNAs for which a cross-build mapping was unavailable (e.g. a coordinate that failed to lift over), contributing to one build's count but not the other. The direction of these single-build entries reflects each database's native assembly: hg19-native resources (Arraystar, circRNADb, circBank, circBase) retain unmapped coordinates as hg19-only, whereas hg38-native resources (circAtlas, CIRCpedia, CSCD, exoRBase) retain them as hg38-only. Second, multiple coordinates in the source build may converge to the same coordinate in the target build after liftover (Multi-mapped), typically where circRNAs fall within segmentally duplicated or repeat-rich loci. This means the number of unique coordinates in the target build is lower than the number of rows in the origin build. All such multi-mappings are retained rather than collapsed, to avoid discarding genuine alternative placements. The small differences between the hg19 and hg38 counts arise from entries mapped in only one build together with a handful of one-to-many coordinate mappings, and do not affect lookups.

Worked example for circRNADb of hg19 origin: 32,914 unique hg19 records. 38 failed to map to hg38 (32,876 one-to-one mappings), with 11 records in hg38 mapping to one or more hg19 values. 32,914 - (38 + 11) = 32,865. Worked example for exorBase of hg38 origin: 126,816 unique hg38 records. 378 failed to map to hg19 (126,438 one-to-one mappings), with 78 records in hg19 mapping to one or more hg38 values. 126,816 - (378 + 78) = 126,360.

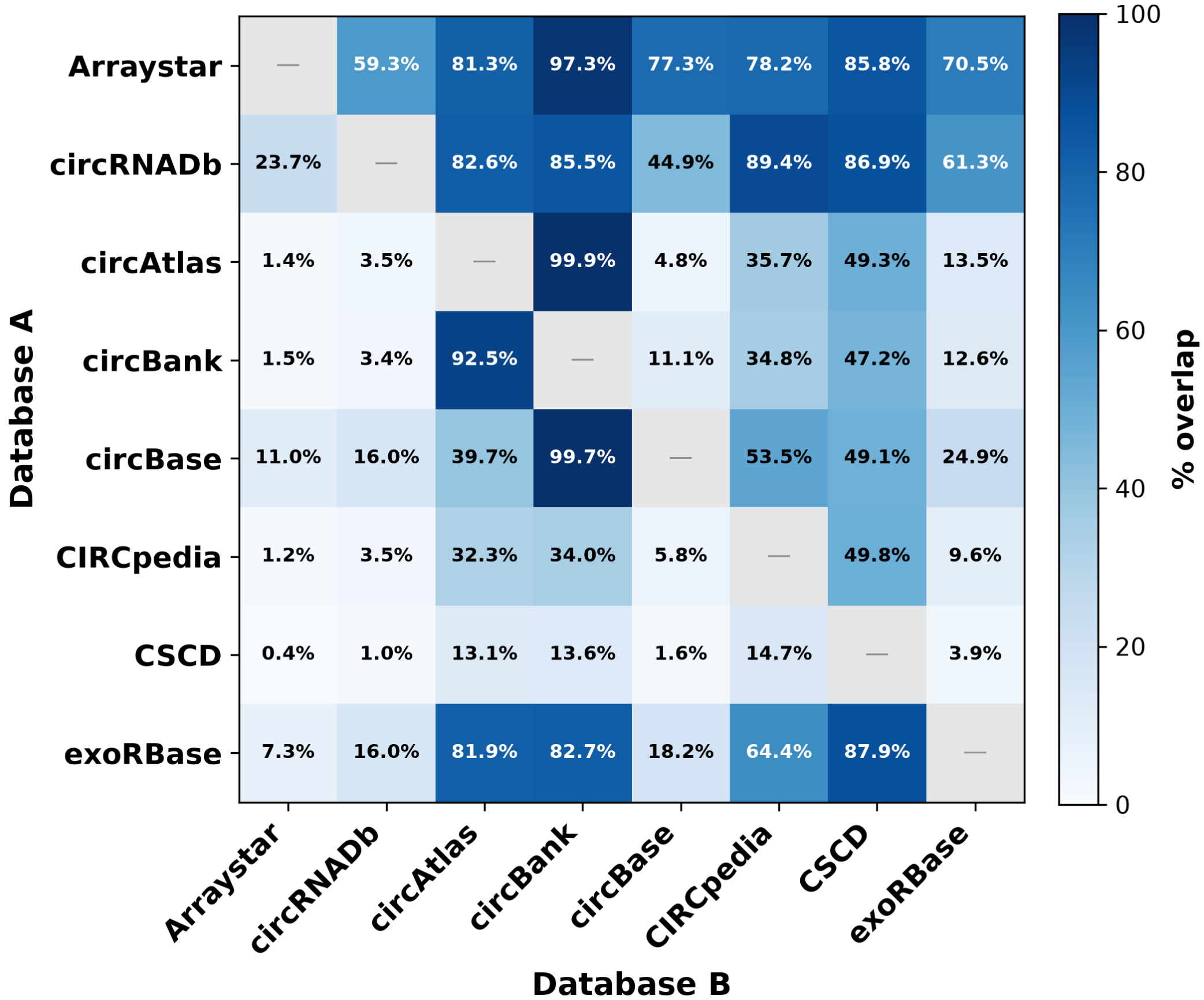

Figure 2: Pairwise hg19 circRNA overlap between pycircdb lookup tables. Each cell gives the percentage of row database A's circRNAs that are also present in column database B.

Table 3: Pairwise circRNA overlap between lookup databases (hg19 coordinates). '% of A' and '% of B' give the share of each database's circRNAs found in the other; Jaccard is the intersection over union.

Database A	Database B	Shared	% of A	% of B	Jaccard
Arraystar	circRNADb	7,800	59.3%	23.7%	20.4%
Arraystar	circAtlas	10,698	81.3%	1.4%	1.4%
Arraystar	circBank	12,798	97.3%	1.5%	1.5%
Arraystar	circBase	10,171	77.3%	11.0%	10.7%
Arraystar	CIRCpedia	10,288	78.2%	1.2%	1.2%
Arraystar	CSCD	11,290	85.8%	0.4%	0.4%
Arraystar	exoRBase	9,269	70.5%	7.3%	7.1%
circRNADb	circAtlas	27,171	82.6%	3.5%	3.5%
circRNADb	circBank	28,130	85.5%	3.4%	3.4%
circRNADb	circBase	14,782	44.9%	16.0%	13.4%
circRNADb	CIRCpedia	29,421	89.4%	3.5%	3.5%
circRNADb	CSCD	28,609	86.9%	1.0%	1.0%
circRNADb	exoRBase	20,170	61.3%	16.0%	14.5%
circAtlas	circBank	766,585	99.9%	92.5%	92.4%
circAtlas	circBase	36,670	4.8%	39.7%	4.5%
circAtlas	CIRCpedia	273,879	35.7%	32.3%	20.4%
circAtlas	CSCD	378,482	49.3%	13.1%	11.6%
circAtlas	exoRBase	103,440	13.5%	81.9%	13.1%
circBank	circBase	92,054	11.1%	99.7%	11.1%
circBank	CIRCpedia	288,436	34.8%	34.0%	20.8%
circBank	CSCD	391,062	47.2%	13.6%	11.8%
circBank	exoRBase	104,528	12.6%	82.7%	12.3%
circBase	CSCD	45,359	49.1%	1.6%	1.5%
circBase	exoRBase	23,004	24.9%	18.2%	11.8%
CIRCpedia	CSCD	422,104	49.8%	14.7%	12.8%
CIRCpedia	circBase	49,461	53.5%	5.8%	5.6%
circBase	exoRBase	81,386	9.6%	64.4%	9.1%
CSCD	exoRBase	111,023	3.9%	87.9%	3.8%

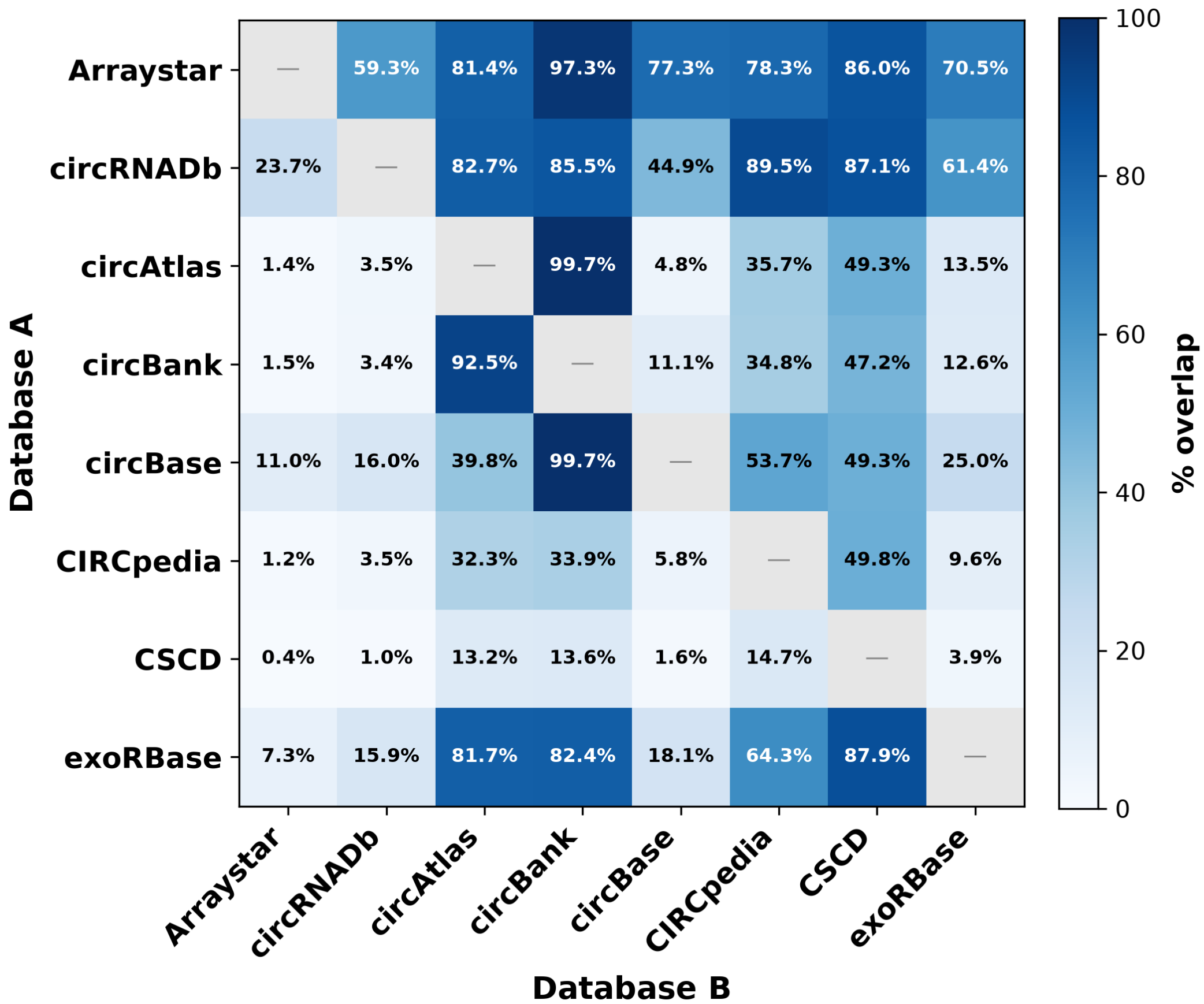

Figure 3: Pairwise hg38 circRNA overlap between pycircdb lookup tables. Each cell gives the percentage of row database A's circRNAs that are also present in column database B.

Table 4: Pairwise circRNA overlap between lookup databases (hg38 coordinates). '% of A' and '% of B' give the share of each database's circRNAs found in the other; Jaccard is the intersection over union.

Database A	Database B	Shared	% of A	% of B	Jaccard
Arraystar	circRNADb	7,796	59.3%	23.7%	20.4%
Arraystar	circAtlas	10,698	81.4%	1.4%	1.4%
Arraystar	circBank	12,786	97.3%	1.5%	1.5%
Arraystar	circBase	10,159	77.3%	11.0%	10.7%
Arraystar	CIRCpedia	10,289	78.3%	1.2%	1.2%
Arraystar	CSCD	11,306	86.0%	0.4%	0.4%
Arraystar	exoRBase	9,271	70.5%	7.3%	7.1%
circRNADb	circAtlas	27,169	82.7%	3.5%	3.5%
circRNADb	circBank	28,112	85.5%	3.4%	3.4%
circRNADb	circBase	14,767	44.9%	16.0%	13.4%
circRNADb	CIRCpedia	29,421	89.5%	3.5%	3.4%
circRNADb	CSCD	28,637	87.1%	1.0%	1.0%
circRNADb	exoRBase	20,171	61.4%	15.9%	14.5%
circAtlas	circBank	766,320	99.7%	92.5%	92.2%
circAtlas	circBase	36,667	4.8%	39.8%	4.4%
circAtlas	CIRCpedia	274,268	35.7%	32.3%	20.4%
circAtlas	CSCD	379,350	49.3%	13.2%	11.6%
circAtlas	exoRBase	103,636	13.5%	81.7%	13.1%
circBank	circBase	91,765	11.1%	99.7%	11.1%
circBank	CIRCpedia	288,310	34.8%	33.9%	20.7%
circBank	CSCD	391,068	47.2%	13.6%	11.8%
circBank	exoRBase	104,492	12.6%	82.4%	12.3%
circBase	CIRCpedia	49,458	53.7%	5.8%	5.5%
circBase	CSCD	45,432	49.3%	1.6%	1.5%
circBase	exoRBase	23,004	25.0%	18.1%	11.7%
CIRCpedia	CSCD	423,292	49.8%	14.7%	12.8%
CIRCpedia	exoRBase	81,576	9.6%	64.3%	9.1%
CSCD	exoRBase	111,477	3.9%	87.9%	3.8%

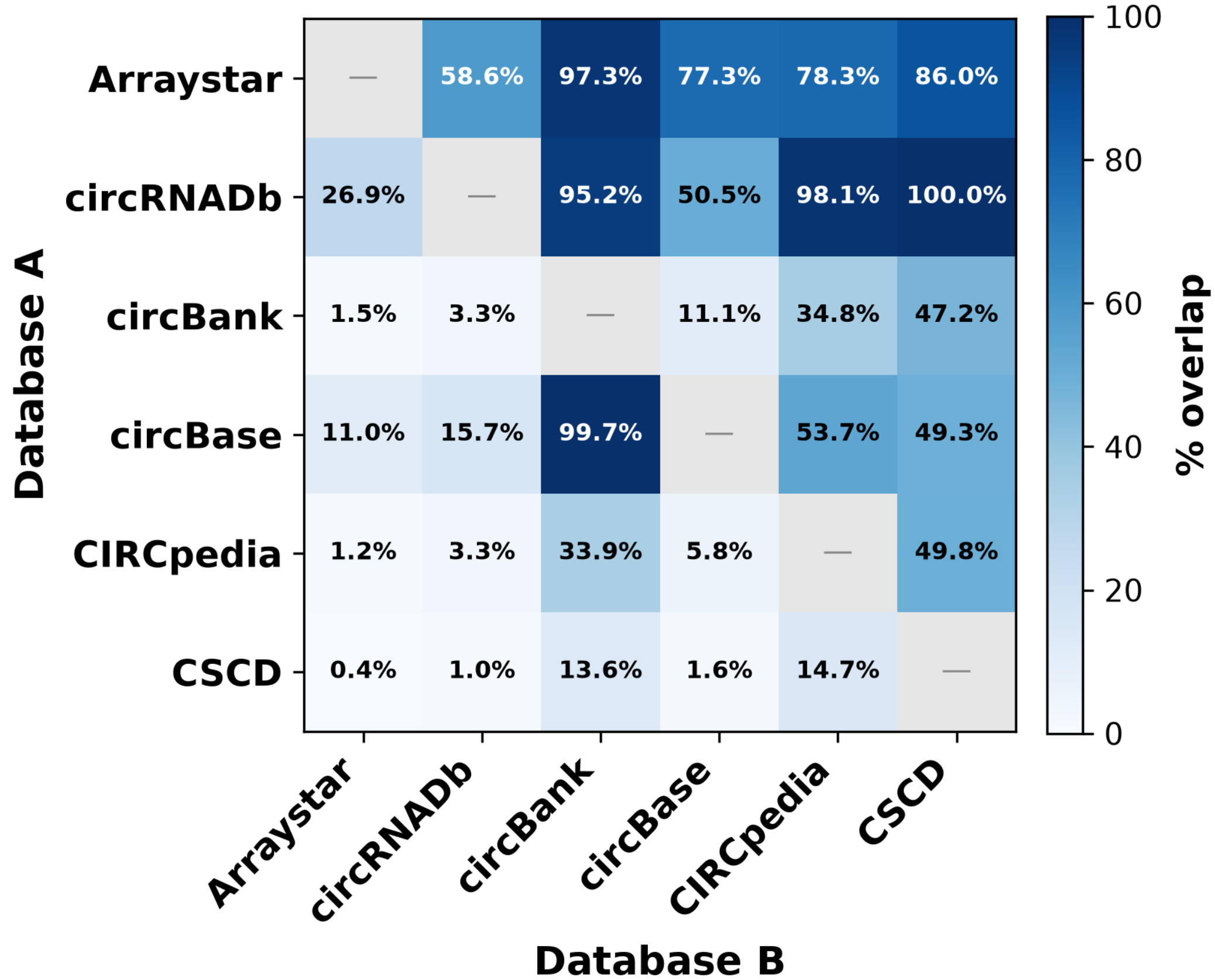

Figure 4: Pairwise coordinate-level overlap among databases with available FASTA sequences (hg38 coordinates). Each cell gives the percentage of row database A's circRNAs that share an hg38 coordinate with column database B. Note: overlap is assessed at the coordinate level, not by sequence identity.

Table 5: Pairwise coordinate-level overlap among databases with available FASTA sequences (hg38 coordinates). Sequence identifiers were mapped to hg38 via each database's lookup table; overlap is assessed at the coordinate level, not by sequence identity. 'Shared' is the number of circRNAs sharing an hg38 coordinate between databases; '% of A'/'% of B' are that count relative to each database; Jaccard is intersection over union.

Database A	Database B	Shared	% of A	% of B	Jaccard
Arraystar	circRNADb	7,703	58.6%	26.9%	22.6%
Arraystar	circBank	12,786	97.3%	1.5%	1.5%
Arraystar	circBase	10,159	77.3%	11.0%	10.7%
Arraystar	CIRCpedia	10,289	78.3%	1.2%	1.2%
Arraystar	CSCD	11,306	86.0%	0.4%	0.4%
circRNADb	circBank	27,263	95.2%	3.3%	3.3%
circRNADb	circBase	14,463	50.5%	15.7%	13.6%
circRNADb	CIRCpedia	28,093	98.1%	3.3%	3.3%
circRNADb	CSCD	28,650	100.0%	1.0%	1.0%
circBank	circBase	91,765	11.1%	99.7%	11.1%
circBank	CIRCpedia	288,310	34.8%	33.9%	20.7%
circBank	CSCD	391,068	47.2%	13.6%	11.8%
circBase	CIRCpedia	49,458	53.7%	5.8%	5.5%
circBase	CSCD	45,432	49.3%	1.6%	1.5%
CIRCpedia	CSCD	423,292	49.8%	14.7%	12.8%

Please note that comparisons between sequences were assessed at the hg38 coordinate level, not the sequence level. The FASTA sequences themselves will differ greatly given that Arraystar, circRNADb, circBank, and circBase are hg19-based, whilst CIRCpedia and CSCD are hg38-based.

Table 6a: Database identifiers returned by pycircdb for six circRNAs present in all eight annotation databases (hg19 coordinates, 0-based), part 1 of 2. The *ARID1A* circRNA corresponds to the worked example in Section 1 of the main manuscript. All identifiers were verified against the downloaded database files.

Database	chr1:27087346 −27089776|+	chr1:100459092 −100464971|+	chr1:100515464 −100535241|+
Gene	ARID1A	SLC35A3	HIAT1
Arraystar	ASCRP3003018	ASCRP3010801	ASCRP3013340
circAtlas	hsa-ARID1A_0002	hsa-SLC35A3_0011	hsa-MFSD14A_0001
circBank	hsa_ARID1A_0003300	hsa_SLC35A3_0000400	hsa_HIAT1_0002000
circBase	hsa_circ_0006913	hsa_circ_0013298	hsa_circ_0000096
CIRCpedia	CIRCHSA_ARID1A_2	CIRCHSA_SLC35A3_1	CIRCHSA_ENSG00000283761_1
circRNADb	hsa_circ_09021	hsa_circ_07782	hsa_circ_25841
exoRBase	exo_circ_001879	exo_circ_005667	exo_circ_005686
CSCD (hg38)	chr1:26760855 −26763285|+	chr1:99993536 −99999415|+	chr1:100049908 −100069685|+

Table 6b: Database identifiers returned by pycircdb for six circRNAs present in all eight annotation databases, part 2 of 2.

Database	chr1:100889777 −100908552|+	chr1:101372407 −101379362|+	chr1:101372407 −101387397|+
Gene	CDC14A	SLC30A7	SLC30A7
Arraystar	ASCRP3001224	ASCRP3004745	ASCRP3010903
circAtlas	hsa-CDC14A_0001	hsa-SLC30A7_0008	hsa-SLC30A7_0001
circBank	hsa_CDC14A_0004500	hsa_SLC30A7_0001600	hsa_SLC30A7_0001800
circBase	hsa_circ_0000097	hsa_circ_0013339	hsa_circ_0000098
CIRCpedia	CIRCHSA_CDC14A_1	CIRCHSA_SLC30A7_5	CIRCHSA_SLC30A7_1
circRNADb	hsa_circ_02823	hsa_circ_16037	hsa_circ_11632
exoRBase	exo_circ_005756	exo_circ_005783	exo_circ_005785
CSCD (hg38)	chr1:100424221 −100442996|+	chr1:100906851 −100913806|+	chr1:100906851 −100921841|+

Table 7: Breakdown of miRNA database containing 184,125,383 circRNA-miRNA pairs. CSCD provides the majority of entries: 177,600,389, with CircNet supplementing 6,913,950. 388,956 miRanda entries are present in both sources; for these overlapping entries, the miRanda score and minimum free energy are taken from CSCD.

Algorithm	circRNA-miRNA
TargetScan	94,435,439
miRanda	45,090,877
miRanda,TargetScan	39,447,094
PITA	4,358,990
miRanda,PITA	667,676
miRanda,PITA,TargetScan	72,601
PITA,TargetScan	52,706

References

[1] Wanying Wu, Peifeng Ji, and Fangqing Zhao. CircAtlas: an integrated resource of one million highly accurate circular RNAs from 1070 vertebrate transcriptomes. *Genome Biol.*, 21(1):1–14, December 2020.

[2] Petar Glažar, Panagiotis Papavasileiou, and Nikolaus Rajewsky. circBase: a database for circular RNAs. *RNA*, September 2014.

[3] Ming Liu, Qian Wang, Jian Shen, Burton B. Yang, and Xiangming Ding. Circbank: a comprehensive database for circRNA with standard nomenclature. *RNA Biol.*, 16(7):899, July 2019.

[4] Dawood B. Dudekula, Amaresh C. Panda, Ioannis Grammatikakis, Supriyo De, Kotb Abdelmohsen, and Myriam Gorospe. CircInteractome: A web tool for exploring circular RNAs and their interacting proteins and microRNAs. *RNA Biol.*, 13(1):34–42, 2016.

[5] Yigang Chen, Lantian Yao, Yun Tang, Jhih-Hua Jhong, Jingting Wan, Jingyue Chang, Shidong Cui, Yijun Luo, Xiaoxuan Cai, Wenshuo Li, Qi Chen, Hsi-Yuan Huang, Zhuo Wang, Weiming Chen, Tzu-Hao Chang, Fengxiang Wei, Tzong-Yi Lee, and Hsien-Da Huang. CircNet 2.0: an updated database for exploring circular RNA regulatory networks in cancers. *Nucleic Acids Res.*, 50(D1):93–101, January 2022.

[6] Xianwen Meng, Dahui Hu, Peijing Zhang, Qi Chen, and Ming Chen. CircFunBase: a database for functional circular RNAs. *Database (Oxford).*, 2019:baz003., January 2019.

[7] Rui Dong, Xu-Kai Ma, Guo-Wei Li, and Li Yang. CIRCpedia v2: An Updated Database for Comprehensive Circular RNA Annotation and Expression Comparison. *Genomics Proteomics Bioinformatics*, 16(4):226–233, August 2018.

[8] Jing Feng, Wenbo Chen, Xin Dong, Jun Wang, Xiangfei Mei, Jin Deng, Siqi Yang, Chenjian Zhuo, Xiaoyu Huang, Lin Shao, Rongyu Zhang, Jing Guo, Ronghui Ma, Juan Liu, Feng Li, Ying Wu, Leng Han, and Chunjiang He. CSCD2: an integrated interactional database of cancer-specific circular RNAs. *Nucleic Acids Res.*, 50(D1):D1179–D1183, January 2022.

[9] Xiaoping Chen, Ping Han, Tao Zhou, Xuejiang Guo, Xiaofeng Song, and Yan Li. circRNADb: A comprehensive database for human circular RNAs with protein-coding annotations. *Sci. Rep.*, 6(34985):1–6, October 2016.

[10] Hang Ruan, Yu Xiang, Junsuk Ko, Shengli Li, Ying Jing, Xiaoyu Zhu, Youqiong Ye, Zhao Zhang, Tingting Mills, Jing Feng, Chun-Jie Liu, Ji Jing, Jin Cao, Bingying Zhou, Li Wang, Yubin Zhou, Chunru Lin, An-Yuan Guo, Xi Chen, Lixia Diao, Wenbo Li, Zhiao Chen, Xianghuo He, Gordon B. Mills, Michael R. Blackburn, and Leng Han. Comprehensive characterization of

circular RNAs in ˜ 1000 human cancer cell lines. *Genome Med.*, 11(1):1–14, December 2019.

[11] Dongxia Yao, Lei Zhang, Mengyue Zheng, Xiwei Sun, Yan Lu, and Pengyuan Liu. Circ2Disease: a manually curated database of experimentally validated circRNAs in human disease. *Sci. Rep.*, 8(11018):1–6, July 2018.

[12] Chunyan Fan, Xiujuan Lei, Jiaojiao Tie, Yuchen Zhang, Fang-Xiang Wu, and Yi Pan. CircR2Disease v2.0: An Updated Web Server for Experimentally Validated circRNA–disease Associations and Its Application. *Genomics Proteomics Bioinformatics*, 20(3):435–445, June 2022.

[13] Suman Ghosal, Shaoli Das, Rituparno Sen, Piyali Basak, and Jayprokas Chakrabarti. Circ2Traits: a comprehensive database for circular RNA potentially associated with disease and traits. *Front. Genet.*, 4:68415, December 2013.

[14] Zhi-Yan Sun, Chang-Lin Yang, Li-Jie Huang, Zong-Chao Mo, Ke-Nan Zhang, Wen-Hua Fan, Kuan-Yu Wang, Fan Wu, Ji-Guang Wang, Fan-Lin Meng, Zheng Zhao, and Tao Jiang. circRNADisease v2.0: an updated resource for high-quality experimentally supported circRNA-disease associations. *Nucleic Acids Res.*, page gkad949, October 2023.

[15] Ling-Ling Zheng, Jun-Hao Li, Jie Wu, Wen-Ju Sun, Shun Liu, Ze-Lin Wang, Hui Zhou, Jian-Hua Yang, and Liang-Hu Qu. deepBase v2.0: identification, expression, evolution and function of small RNAs, LncRNAs and circular RNAs from deep-sequencing data. *Nucleic Acids Res.*, 44(D1):196–202, January 2016.

[16] Hongyan Lai, Yuchen Li, Hena Zhang, Jia Hu, Jiatao Liao, Ying Su, Qin Li, Bing Chen, Caiping Li, Zhen Wang, Yan Li, Jialei Wang, Zhiqiang Meng, Zhaohui Huang, and Shenglin Huang. exoRBase 2.0: an atlas of mRNA, lncRNA and circRNA in extracellular vesicles from human biofluids. *Nucleic Acids Res.*, 50(D1):118–128, January 2022.

[17] Josh N. Vo, Marcin Cieslik, Yajia Zhang, Sudhanshu Shukla, Lanbo Xiao, Yuping Zhang, Yi-Mi Wu, Saravana M. Dhanasekaran, Carl G. Engelke, Xuhong Cao, Dan R. Robinson, Alexey I. Nesvizhskii, and Arul M. Chinnaiyan. The Landscape of Circular RNA in Cancer. *Cell*, 176(4):869–88113, February 2019.

[18] Kieran Walsh, Akira Gokool, Hamid Alinejad-Rokny, and Irina Voineagu. NeuroCirc: an integrative resource of circular RNA expression in the human brain. *Bioinformatics*, 37(20):3664–3666, October 2021.

[19] Jun-Hao Li, Shun Liu, Hui Zhou, Liang-Hu Qu, and Jian-Hua Yang. starBase v2.0: decoding miRNA-ceRNA, miRNA-ncRNA and protein–RNA interaction networks from large-scale CLIP-Seq data. *Nucleic Acids Res.*, 42(D1):D92–D97, January 2014.

[20] Zhidong Tang, Xuecang Li, Jianmei Zhao, Fengcui Qian, Chenchen Feng, Yanyu Li, Jian Zhang, Yong Jiang, Yongsan Yang, Qiuyu Wang, and Chunquan Li. TRCirc: a resource for transcriptional regulation information of circRNAs. *Briefings Bioinf.*, 20(6):2327–2333, November 2019.
